# Center of pressure based segment inertial parameters validation

**DOI:** 10.1371/journal.pone.0180011

**Published:** 2017-06-29

**Authors:** Clint Hansen, Nasser Rezzoug, Philippe Gorce, Brice Isableu, Gentiane Venture

**Affiliations:** 1 Christian-Albrechts University, Faculty of Medicine, Department of Neurology, Kiel, Germany; 2 University of Toulon, HandiBio, Toulon, France; 3 Aix Marseille Univ, PSYCLE, Aix-en-Provence, France; 4 Tokyo University of Agriculture and Technology GVLab, Tokyo, Japan; Universite de Nantes, FRANCE

## Abstract

By proposing efficient methods for estimating Body Segment Inertial Parameters’ (BSIP) estimation and validating them with a force plate, it is possible to improve the inverse dynamic computations that are necessary in multiple research areas. Until today a variety of studies have been conducted to improve BSIP estimation but to our knowledge a real validation has never been completely successful. In this paper, we propose a validation method using both kinematic and kinetic parameters (contact forces) gathered from optical motion capture system and a force plate respectively. To compare BSIPs, we used the measured contact forces (Force plate) as the ground truth, and reconstructed the displacements of the Center of Pressure (COP) using inverse dynamics from two different estimation techniques. Only minor differences were seen when comparing the estimated segment masses. Their influence on the COP computation however is large and the results show very distinguishable patterns of the COP movements. Improving BSIP techniques is crucial and deviation from the estimations can actually result in large errors. This method could be used as a tool to validate BSIP estimation techniques. An advantage of this approach is that it facilitates the comparison between BSIP estimation methods and more specifically it shows the accuracy of those parameters.

## Introduction

Biomechanical and clinical human movement analyses contain a variety of measurements to evaluate the performance or the health status of subjects. Generally, those measurements are based on mathematical and physical models that are applied to the human body. Among them, body segment inertial parameters (BSIPs) (which are: mass, center of mass (COM), and inertia tensor) have been shown to be highly important for clinical and biomechanical research [1,2]. They allow monitoring the variations in muscle-mass in patients during hospitalization, rehabilitation, or neurological examination [3] and are of crucial importance for biomechanical analyses such as inverse dynamics computations [4–6], in particular for gait analyses.

Even though the BSIP estimation methods have improved recently, most of the regression models still show estimation error when applied beyond the sample population. As an example, models based on regression equations do not adjust for extreme populations as children, pregnant women, or people with a minor health status issue and mostly assume symmetric bodies. Ongoing research has revealed differences in the BSIP of different ethnic groups and some anthropometric tables were adjusted accordingly [7]. The BSIP estimation has been improved over time by adapting segment lengths [1,8–10], using geometric models based on numerous anthropometric measurements [7,11], taking results from cadavers’ studies [12–14], and in vivo mass-scanning based models on living subjects [15]. Regardless of the improvements, they remain regression methods using scaling functions [16] based on earlier collected databases e.g. [17], and fail in providing personalized data.

Also, various BSIP estimation techniques use density values from the literature that may not correspond to the actual samples. Image-guided methods as MRI or DEXA scans [18–21] do have the ability to provide those values, unfortunately these approaches are expensive and can be associated with radiation exposure.

Even though the image-guided methods could provide high quality BSIP estimation, their validity remains to be established and only few studies have evaluated them [22]. In contrast, mathematical models based on identification have been proposed to validate the estimated parameters against experimental measurements [23–28]. In general, a force plate serves as the validation tool [29]. The theoretical or modeled parameters are compared to the measured ground reaction forces [23] or the center of pressure (COP, the point of application of the vertical resultant force acting on the body from the supporting surface) movement.

Some researchers investigated the possibility of estimating specific segment masses of subjects using a force plate [29,30] and others matched the measured external moments with the rate of change of the body’s angular momentum [31]. Also, the vertical GRF and the COP were predicted and compared with the real measurement during static postures, squatting, and level walking [23]. Although methods based on identification have proven to be accurate, the validity of BSIP estimation depends heavily on the type of executed movements during the identification process. For validation purposes, previous research has considered only one activity such as overarm throwing, squat, or even the same movement as executed during the identification. Therefore, in order, to obtain a more thorough cross validation, it is proposed to assess the accuracy of BSIP identification by considering various movements involving lower limbs such as walking, running, or upper-limb, and whole body movements as overarm throwing and basketball throws. To highlight the improvements due to identification, two BSIP estimation methods will be considered: the first one is based on identification through optimization (OM) [32,33] and the second one is regression-based (RM) [8]. Both are non-invasive and, radiation-free. Their accuracy will be evaluated by comparing the body segment masses and reprocessed and measured COP data during a variety of movements. The novelty of this article is the statistical comparison of the segment masses, the additional cross validation with multiple short movements and comparison with the literature.

## Methods

### Human model

**Fig 1:**
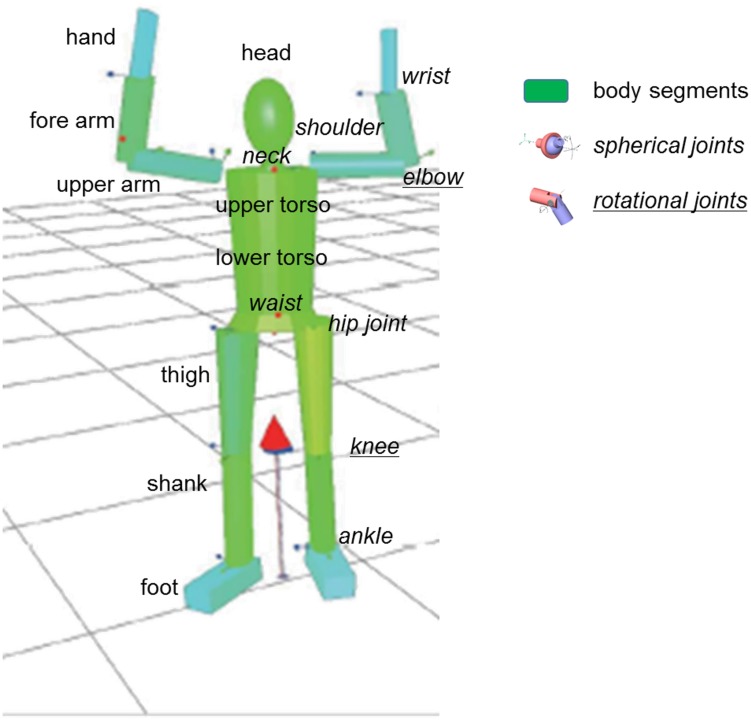
The visualized human body model including the markers attached to body and the ground reaction force represented as a vector.

To obtain accurate identification results of the BSIPs it is important to define a kinematic model to describe the human body and to obtain its characteristic geometric parameters. We consider a model of the human body with 34 degree of freedom (DOF) and 15 rigid links [32–34]: upper torso, lower torso, head, upper arms, fore arms, hands, thighs, shanks, and feet (Fig 1). The waist, the neck, the shoulders, the wrists, the hip joints and the ankles are modeled with spherical joints, and the elbows and the knees are modeled with rotational joints following the recommendations of Venture [32].

### Inverse kinematics (IK) and Kinetic model

From the position of reflective markers attached to the subjects, the joint angles of the considered model are obtained by inverse kinematics [3] [35].

A Newton—Euler inverse dynamics algorithm [36,37] estimates the net torques of all the anatomical structures across a joint necessary to rotate the body segments during the considered tasks. The equation can be written as Eq (1):

$$\tau =H\left(q\right)\ddot{q}+c\left(q,\;\dot{q},fext\right)$$ (1)

with ***q***, $\dot{q}$, $\ddot{q}$ and ***τ*** as the joint-space position, velocity, acceleration and torque and **H** is the generalized inertia matrix, c is the bias force vector including centrifugal, Coriolis and gravity forces.

### Body segment inertia estimation

In the following, both the regression (RM) as well as the optimization based methods (OM) are briefly detailed.

#### Regression based method

The considered BSIPs of each segment were: the mass, the position of the center of mass (COM) relative to the segment proximal joint coordinate frame and the inertia tensor at the segment COM expressed in the coordinate system of the segment. They were assessed from the subject’s mass and segment length obtained by the optolectronic system according to [4].

The estimation of the BSIP is based on the scaling functions proposed by [1] that adjust the data of [17] and of [38]. They are expressed directly in the conventional segment coordinate systems (SCS) and do not restrain the position of the COM and the orientation of the principal axes of inertia. Given the body mass of a subject and the appropriate scaling, one can estimate the segment mass (*m*) of each segment (*i)*. Given the specific scaling factors and the length of the segments (*L*_*i*_), the position of the segment COM in the local frame can be defined by using the following equation Eq (2):

$$g_{i}\;=L_{i}{\left[\begin{array}{ccc} X\left(\%\right) & Y\left(\%\right) & Z\left(\%\right) \end{array}\right]}^{T}$$ (2)

*g*_*i*_ denotes the position of the COM of segment *i*.

Based on the segment mass and length and the appropriate scaling factors, the inertia tensor of each segment in its local frame located at the COM can be calculated following Eq (3)

$$I_{ij}=m_{i}{\left(r_{jk}L_{i}\right)}^{2}$$ (3)

*m*_*i*_ and *L*_*i*_ denote the mass and length of segment *i*, respectively,

*r*_*jk*_ (*j* = X, Y, and Z, *k* = X, Y, and Z) are the scaling factors obtained from [1].

#### Optimization based method (OM)

The OM calculates the mass, the COM and anti-symmetric matrix of inertia including the moments and products of inertia of each predefined segment. The inertial parameters are calculated using a least squares method from the external forces and positions of each body segment, based on the general equation of motion for bipedal systems.

We briefly recall the method as it has been introduced previously [26]. Inverse dynamics for a bipedal system are given by Eq (4). The upper part of the model represents the free motion of the base-link (usually chosen as the lower abdomen) while the lower part represents the motions of the N bodies of the various kinematic chains constituting the whole-body. N depends on the anatomy and the complexity chosen for the model.

$$\left[\begin{array}{cc} H_{11} & H_{12}\\ H_{21} & H_{22} \end{array}\right]\left[\begin{array}{c} {\ddot{q}}_{0}\\ \ddot{\theta } \end{array}\right]+\left[\begin{array}{c} b_{1}\\ b_{2} \end{array}\right]=\;\left[\begin{array}{c} 0\\ \tau \end{array}\right]+\;\sum_{k=1}^{n_{c}}\left[\begin{array}{c} K_{k1}\\ K_{k2} \end{array}\right]F_{k},$$ (4)

where:

- **H**_**ij**_ (i, j = 1, 2) is the appropriate inertia matrix,
- ${\ddot{q}}_{0}$ is the vector of generalized coordinates acceleration which represents the 6 DOF of the base-link,
- $\ddot{\theta }$ is the vector joint angular accelerations,
- **b**_**i**_ is the bias force vector including centrifugal, Coriolis and gravity forces,
- **τ** is the vector of joint torques,
- *N*_*c*_ is the number of contact points with the environment,
- **F**_**k**_ is the k th vector of external forces exerted on the human body,
- **K**_**k1**_ and **K**_**k2**_ are matrices which map **F**_**k**_ to the generalized force vector.

As shown in [8] and [13], the inverse dynamics can be expressed in a linear form with respect to the dynamic parameters. Thus, by separating the vector of inertial parameters **ϕ** from the observation matrix Y the identification model is obtained in Eq (5).

$$\left[\begin{array}{c} Y_{1}\\ Y_{2} \end{array}\right]\phi =\;\left[\begin{array}{c} 0\\ \tau \end{array}\right]+\;\sum_{k=1}^{n_{c}}\left[\begin{array}{c} K_{k1}\\ K_{k2} \end{array}\right]F_{k},$$ (5)

$Y=\left[\begin{array}{c} Y_{1}\\ Y_{2} \end{array}\right]$ is the observation matrix or regressor. It is a function of the model displacement, velocities and accelerations. The vector of inertial parameters **Φ** = [**Φ**_1_,…, **Φ**_n_] can be written as **ϕ**_**i**_ = [*m*_*i*_ *ms*_*i*,*x*_ *m*_*si*,*y*_ *m*_*si*,*z*_ *I*_*i*,*xx*_ *I*_*i*,*yy*_ *I*_*i*,*zz*_ *I*_*i*,*yz*_ *I*_*i*,*zx*_ *I*_*i*,*zy*_]^*T*^; *m*_*i*_ is the mass, *I*_*i*,*xx*_, *I*_*i*,*yy*_, *I*_*i*,*zz*_, *I*_*i*,*yz*_, *I*_*i*,*zx*_, *I*_*i*,*zy*_, are the six independent components of the inertia matrix *I*_*i*_, *ms*_*i*,*x*_ *ms*_*i*,*y*_ *ms*_*i*,*z*_ are the first moments components of the vector *ms*_*i*_.

$$Y_{B1}\phi =\left[\begin{array}{c} Y_{B1}\\ Y_{B2} \end{array}\right]\phi_{B}=\sum_{k=1}^{N_{c}}\left[\begin{array}{c} K_{k1}\\ K_{k2} \end{array}\right]F_{k}$$ (6)

To identify the inertial parameters, the mathematical structure of the minimal identification model offers the possibility to identify ϕ considering only the upper part of Eq (6). After sampling along the motion, the system of equations given in Eq (7) is solved by the least squares method.

$$Y_{B1}\phi =\sum_{k=1}^{n_{c}}K_{k1}F_{k}$$ (7)

Based on the BSIPs and the kinematic chain movements, the time series of ground reaction forces (GRF) components can be computed and compared with the measured GRFs with the force plate. In addition to the three forces, the three moments of the force plate were calculated to analyze the COP movement during the experiments. The COP position was computed using Eqs (8) and (9):

$$COP_{ML}=\;\left(\frac{-M_{ML}+\;F_{AP}\times d}{F_{z}}\right)$$ (8)

$$COP_{AP}=\;\left(\frac{M_{AP}+\;F_{ML}\times d}{F_{z}}\right)$$ (9)

where F stands for force, M for moment of force, F_Z_ is the vertical component of the GRF, d is the distance between the surface of the platform and its origin, each in the medio-lateral (ML) and anterior-posterior (AP) direction. Also, this study evaluates the effect of incorrect BSIP estimation between the calculated and originally obtained COP.

#### Subjects

Twelve male subjects (age: 22 ± 4 years height: 162.7±5.01 cm, weight: 62.8±8.96 kg) voluntarily participated in the experiment after signing a statement of informed consent as required by the Helsinki declaration and the local Ethics Committee of the University Paris-Saclay EA 4532 (http://www.staps.u-psud.fr/fr/recherche/comite-ethique-local.html), who specifically approved this study. For the supplementary online video, we obtained the subjects' specific consent for publication.

#### Experiment

Subjects performed a 120 second predefined sequence of movements on the force plate which involves a variety of movements trying to use each DOF of the body with a range of angular velocities and accelerations [22]. The sequence was demonstrated to the subjects by a video that was shown multiple times. During the performance, subjects had the video projected in front of them to ensure a standardized movement on the force plate (for additional information please see the supplementary video online).

#### Cross validation

To evaluate the impact of the BSIPs estimation during discrete movements, additional test conditions were employed including walking, running, over arm throwing, and a basketball throw. The motions were chosen as they have a limited contact time with the force plate and involve high segment accelerations [26].

#### Motion capture and markerset

The motions were recorded at a frequency of 250 Hz by an optical motion capture system consisting of eight cameras (T160 series, Vicon motion systems Inc., Oxford, UK). Reflective markers were attached to the body of the subjects. These markers were located on defined bony landmarks to insure accuracy of the inverse kinematics computations due to reduced skin movement artifacts. Participants wore thirty five markers according to the following anatomic landmarks: right and left temple, front of the head, 7th cervical vertebrae, 10th thoracic vertebrae, xiphoid process, notch where the clavicles meets the sternum, middle of the right scapula, right and left anterior superior iliac spine, right and left posterior superior iliac spine, right and left acromio-clavicular joints, right and left upper arm, lateral epicondyle of the right and left elbow, right and left forearm, right and left side of the wrist joint of the right and left wrist, right and left hand, medial and lateral epicondyle of the right and left knee, medial and lateral epicondyle of the right and left malleolus, the back of the calcaneus of the left and right foot and the 2nd metatarsal of the left and right foot. The anthropometric parameters of the human body for each segment were measured automatically using the passive optical reflective marker positions. The contact forces were measured by one force-plate (Bertec Corporation, Columbus, OH, USA) at 1000 Hz.

## Data analysis

### Body segment mass

The estimated body segment masses were compared to each other to understand if both estimation techniques differ in terms of mass estimation.

### COP computations compared to the force plate

To evaluate the differences between the computed and real measurements made with the force plate the Root-Mean Square Error (RMSE) between the calculated (OM and RM) and measured COP was determined for each subject.

### Method comparison based on posturographic analysis parameter

To quantify differences between the measured and the reconstructed (OM and RM) force plate data, the following variables were calculated: (1) the root-mean square (RMS) of COP excursion on anterior—posterior (RMS_AP_) and medial—lateral (RMS_ML_) axes [mm]; (2) mean body sway velocity (MV), calculated as the first time derivative of COP AP and ML displacement [mm/s]; (3) sway length (SL), length of COP trajectory displacements on the platform surface [mm]; and (4) surface area (SA): area containing 95% of COPs [mm2].

**Fig 2:**
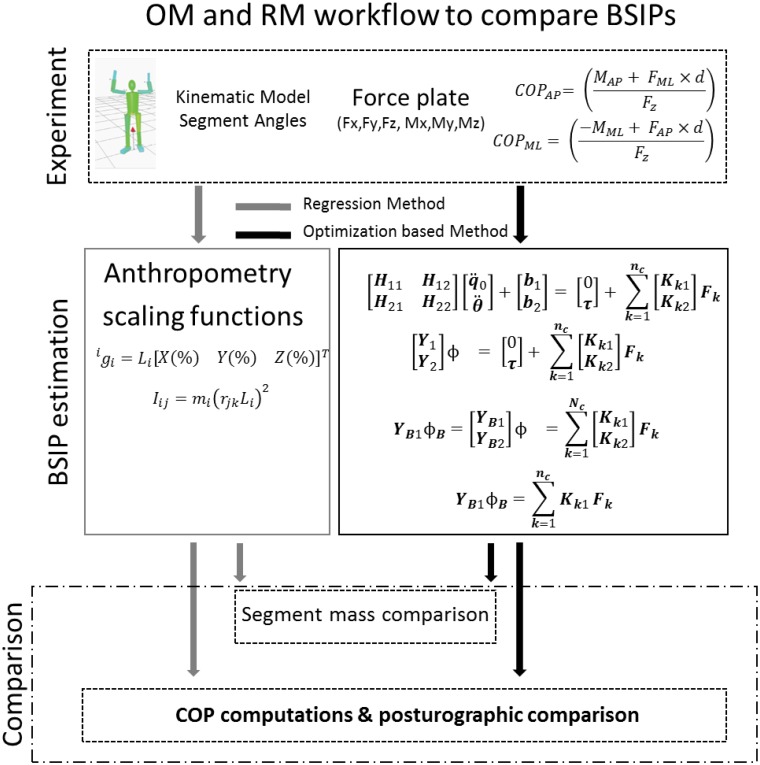
Schematic representation of the workflow and computation of the regression method (RM) and the optimization method (OM).

To visualize the workflow of the conducted study Schematic representation of the workflow and computation of the regression method (RM) and the optimization based method (OM) are shown in Fig 2.

## Statistical analyses

### Segment mass

Following a normality test using Kolmogorov-Smirnov (K-S) tests with Lilliefors significance correction paired samples T-tests were conducted to identify differences in the identified segment masses.

### COP comparisons

Following a normality test using Kolmogorov-Smirnov (K-S) tests with Lilliefors significance correction, the RMSE errors were analyzed in both the AP and ML direction using paired samples T-tests.

### Posturographic analysis

The means of each posturographic variable were determined and following a normality test using the Kolmogorov-Smirnov (K-S) test with Lilliefors significance correction, the data was analyzed using a one-way analysis of variances (ANOVA) to evaluate the estimation techniques (RM and OM) against measured parameters with the force platform (FP). The dependent variables were the variability MV, SL, SA, RMS_AP_, and RMS_ML_. The significance level was set at α = 0.05 for all the statistical tests.

## Results

### Segment mass

Significant differences were found for all parameters except the left upper arm and forearm and the right forearm (Table 1).

**Table 1:** The direct comparison mean(standard deviation)) between the segment mass estimation (kg) of both methods shows no differences except for the left arm, left forearm and the right forearm.

	OM	RM				
	M(SD)	M(SD)	95%CI	t-value	p	Cohen’s d
**lower torso**	8.79(1.10)	8.85(1.11)	[-0.10, -0.01]	-2.79	.018	0.05
**upper torso**	20.62(2.57)	20.75(2.59)	[15.08, 17.80]	26.59	.000	0.05
**head**	4.05(0.46)	4.17(0.52)	[-0.24, -0.01]	-2.44	.033	0.26
**left upper arm**	1.48(0.19)	1.50(0.19)	[-0.13, 0.11]	-0.20	.845*	0.06
**left forearm**	1.07(0.16)	1.06(0.13)	[-0.03, 0.07]	0.70	.499*	-0.11
**left hand**	0.95(0.25)	0.37(0.05)	[0.42, 0.72]	8.28	.000	-3.21
**right upper arm**	2.08(0.34)	1.50(0.19)	[0.43, 0.74]	8.36	.000	-2.12
**right forearm**	1.03(0.15)	1.06(0.13)	[-0.07, 0.01]	-1.70	.117*	0.22
**right hand**	0.54(0.17)	0.37(0.05)	[0.08, 0.26]	4.07	.002	-1.37
**left thigh**	7.14(0.95)	7.66(0.96)	[-0.60, -0.46]	-16.90	.000	0.55
**left shank**	2.60(0.38)	2.99(0.37)	[-0.46, -0.33]	-14.06	.000	1.04
**left foot**	1.10(0.26)	0.75(0.09)	[0.23, 0.48]	6.25	.000	-1.79
**right thigh**	7.21(0.94)	7.66(0.96)	[-0.53, -0.38]	-12.93	.000	0.48
**right shank**	2.67(0.38)	2.99(0.37)	[-0.39, -0.26]	-10.96	.000	0.85
**right foot**	1.02(0.24)	0.75(0.09)	[0.17, 0.38]	5.59	.000	-1.52

* non-significant differences (p>0.05) between segment masses

### COP comparisons

**Fig 3:**
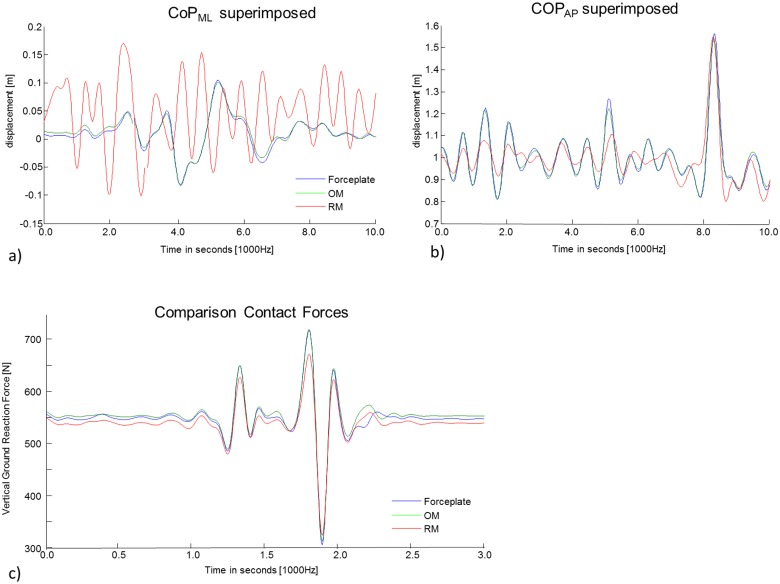
The center of pressure of the original force plate and the reconstructed COP movement using the OM and RM, in a) COP_ML_ and b) COP_AP_ direction are represented for 10 seconds of the 120sec movement trial for a representative subject; c) the force plate and the reconstructed vertical ground reaction forces are represented over the time of a basketball throwing motion of a representative subject.

The paired samples T-tests showed lower error estimation for OM compared to RM in both A/P and M/L direction (p<0.05) (Table 2). To visualize the differences caused by the two estimation methods, Fig 3 shows the measured and computed COP_ML_ and COP_AP_.

**Table 2:** Means and standard deviation (M(SD)) of COP errors [mm] during dynamic procedures calculated using BSIP's obtained from the OM and RM and the force plate (FP).

	OM	RM				
RMSE	M(SD)	M(SD)	95%CI	t-value	p	Cohen’s d
AP	3.84(1.49)	12.69(4.82)	[-91.69, -77.96]	-28.00	.018*	2.48
ML	88.67(11.03)	69.63(22.36)	[-69.30, -44.59]	-10.15	.000*	1.08

* significant differences (p<0.05)

### Posturographic analysis

ANOVA showed significant differences in the RMS_AP_ direction with differences between OM and RM (p<0.05) and force plate and RM (p<0.05). No significant differences were uncovered for the RMS_ML_, MV, SL, and SA (Table 3).

**Table 3:** Means and standard deviation (M(SD)) of posturographic variable during dynamic procedures calculated using BSIP's obtained from the OM and RM and the force plate (FP).

	FP	OM	RM		
	M(SD)	M(SD)	M(SD)	F-value	p
**MV**	6.26(2.70)	6.28(2.60)	6.52(1.76)	0.04	0.96
**SL**	37.11(15.73)	37.24(15.13)	38.68(10.28)	0.04	0.96
**SA**	0.09(0.06)	0.09(0.05)	0.09(0.05)	0.00	1.00
**RMS_AP_**	0.05(0.01)*	**0.05(0.01)**^+^	**0.08(0.01)** ^+^*	30.97	**0.00**
**RMS_ML_**	1.01(0.01)	1.01(0.01)	1.01(0.00)	1.26	0.30

*Bonferroni post-hoc differences between FP and RM;

^+^Bonferroni post-hoc differences between OM and RM p<0.05

### Cross validation

The COP and the GRF of the OM shows higher consistency than the RM when compared to the measured experimental force platform measures (Table 4). When comparing the COP errors, for example, walking OM shows smaller errors compared to the literature and to RM. However, RM seems a valid method when comparing vertical ground reaction forces, but still shows higher errors compared to OM (Table 4). To visualize the differences caused by the two estimation methods, Fig 3c shows the computed vertical ground reaction force during the discrete cross validation movements.

**Table 4:** Means and standard deviation (M(SD)) of COP errors and vertical GRF during dynamic procedures calculated using BSIP's obtained from the current method and RM are compared with results with Chen, 2011.

	Chen 2011	OM	RM
**COP error mm**			
Walking	12.08(2.08)	5.04(0.78)	48.03(4.22)
Running	/	7.95(1.40)	55.54(5.91)
Basketball	/	7.43(1.43)	52.18(2.83)
Random (120s)	/	9.42(3.60)	80.02(16.17)
Throwing	/	4.6(2.4)	15.5(1.2)
**Vertical GRF [% BW]**			
Walking	4.8(1.10)	1.47(1.43)	3.07(1.12)
Running	/	5.64(2.80)	7.48(2.46)
Basketball	/	1.54(0.19)	2.09(0.27)
Random (120s)	/	0.84(0.25)	4.77(1.61)
Throwing	/	2.35 (0.86)	7.51(2.17)

## Discussion

The aim of the present study was to investigate the influence of two BSIP estimation methods on the COP movement during motions, using an optical motion capture system and a force plate. Compared to previous research [23,26] and to obtain a more thorough cross validation, we assessed the accuracy of BSIP identification by considering movements involving walking, running, overarm throwing and basketball throws.

The performance of the OM and the RM was evaluated against real measurements and the statistical analyses have shown a superior performance of the OM compared to the regression method (which based its calculations on general equations and scaling functions of a sample population). Those findings are in accordance with previously discussed approaches [12–14] and OM proves robust without exposing the subjects to radiation [18,19], time intensive anthropometric measurements [7,8] or even invasive procedures.

The segment masses differ with three exceptions (left arm and forearm, and the right forearm), and the hands seem heavier in the OM compared to the RM. Those differences could be a result of the type of executed movements during the identification process. The segment masses of the forearm and hand may have merged while the inter-segment mass distribution stayed constant, so those errors could compensate for each other, making the choice of the kinematic chain and the movement of primary importance.

Identical kinematic input (from the motion capture system) was used for the comparison leading to the assumption that the differences measured by the output only result from the estimated BSIPs differences. Manipulating the BSIPs of a model using the same kinematical input will have an impact on the resulting GRFs and COP movements. This leads to the suggestion that the output of the model provides data for a meaningful quantitative analysis of the performance of different BSIP methods.

The force plate serves as the ground truth as it measures GRFs and COP displacement generated by the participant moving across it. The recorded motion can then be used with the estimated BSIPs to objectively compare estimation methods with the force plate recordings. The differences between the calculated and measured displacements provide a quantitative performance of the BSIP estimation method [23,26].

A characteristic feature is the direct evaluation of the BSIP with the force plate making comparisons between subjects and studies (see Table 4) possible [26,29]. For this reason, we recommend that BSIPs are compared using both the GRFs and the COP.

When working with humans the body segments are often considered as rigid bodies, however soft-tissue artifacts and wobbling masses have a strong effect during biomechanical analyses [39]. Optoelectronic measures always include soft-tissue artefacts that will ultimately influence the markers movements and therefore the inverse kinematics computations [40]. Various methods have been proposed including global optimization, rigid marker clusters, or even bone pins [41]. This research however makes use of a marker set based on bony landmarks and a healthy population without extreme body shapes (over-weight or extremely developed muscle mass) and therefore we consider the effect of the wobbling masses on the dynamics as negligible.

To conclude, a standardized evaluation using a force plate could facilitate the comparison and validation of new and existing estimation techniques making it a help in clinical and biomechanical research. In comparison with the measured ground reaction forces the OM approach has shown advantages compared to models proposed in previous research [1]. Our work does not contradict previous research but recommends comparing BSIPs using a force plate. Further investigations are required to demonstrate how BSIP estimation techniques reduce COP errors when testing either normal population or specific subject cases such as; age, gender, body type, and fitness level.

## Supporting information

S1 File Plos_One_Dumas.m. This file calculates calculate Body Segment Inertial Parameters as introduced in the manuscript and referred to as RM.
(M) Click here for additional data file.

S2 File Mat_Ine.m. This function returns the coefficients of the matrix of inertia in matrix form and is a subfunction of S1.
(M) Click here for additional data file.

S3 File BSIP estimation technique DUMAS detail.pdf. This document explains in detail how the scaling functions apply to calculate the BSIPs of the body.
(PDF)
