# Supplementary material for: Center of pressure based segment inertial parameters validation
Source: PLoS One. 2017 Jun 29;12(6):e0180011. doi: 10.1371/journal.pone.0180011 (PMC5491329; doi:10.1371/journal.pone.0180011)
Supplement: S3 File — This document explains in detail how the scaling functions apply to calculate the BSIPs of the body. (PDF) [file pone.0180011.s003.pdf]

BSIP estimation technique (RM)

The RM of the BSIP is based on the scaling functions proposed by Dumas et al. (2007). The scaling functions adjust the data of McConville et al. (1980) and of Young et al. (1983) and are expressed directly in the conventional segment coordinate systems (SCS) and do not restrain the position of the center of mass and the orientation of the principal axes of inertia. Besides the abovementioned, the method proposed by Dumas et al. (2007) is easy to apply in the laboratory and the subjects are not exposed to radiation kept for a long period time in the laboratory.

On the basis of research results of Dumas et al. (2007), the following subchapter conduces to explain the application of the proposed scaling functions on one male exemplar for the upper limb segments.

Table 1 Scaling factors for the position of the center of mass (Dumas et al., 2007)

Segment	Gender	*mass m (%)*
Arm	M	2.4
	F	2.2
Forearm	M	1.7
	F	1.3
Hand	M	0.6
	F	0.5

Given the whole body mass of a subject and the appropriate scaling one can estimate the segment mass:

m_A is the mass of the arm (A), m_F the mass of the forearm (F), m_H the mass of the hand (H), and m the mass of the subject.

The scaling factors to determine the position of the center of mass (CoM) of segment *i* (*i* = A, F, or H) in its local frame are given in the following table:

Table 2 Scaling factors for the position of the center of mass (Dumas et al., 2007)

Segment	Gender	*X* (%)	*Y* (%)	*Z* (%)
Arm	M	1.7	-45.2	-2.6
	F	-7.3	-45.4	-2.8
Forearm	M	1	-41.7	1.4
	F	2.1	-41.1	1.9
Hand	M	3.5	-35.7	3.2
	F	3.3	-32.7	2.1

Given these factors and the length of the segments (L_i), the position of the segment CoM in the local frame can be defined by using the following equation:

$$ {}^{i}\boldsymbol{g}_{\boldsymbol{i}} = \boldsymbol{L}_{\boldsymbol{i}}\,[\boldsymbol{X}(\%) \quad \boldsymbol{Y}(\%) \quad \boldsymbol{Z}(\%)]^{\boldsymbol{T}} \qquad (9) $$

The scaling factors to define the inertia tensor of each segment in its local frame located at the CoM are given in the following table (*i* denotes the complex number such that i^2= -1). These values are valid for male and female subjects.

Table 3 Scaling factors for tensor of inertia (Dumas et al., 2007).

Segment	Gender	r_{XX} (%)	r_{YY} (%)	r_{ZZ} (%)	r_{XY} (%)	r_{XZ} (%)	r_{YZ} (%)
Arm	M	31	14	32	6	5	2
	F	33	17	33	3	5(i)	14
Forearm	M	28	11	27	3	2	8*i*
	F	26	14	25	10	4	13(i)
Hand	M	26	16	24	9	7	8*i*
	F	41	45	36	15(i)	0	0

Given these scaling factors, the terms of the inertia tensor are obtained by the following equation:

$$I = \begin{bmatrix} I_{xx} & I_{yx} & I_{zx} \\ I_{xy} & I_{yy} & I_{zy} \\ I_{xz} & I_{yz} & I_{zz} \end{bmatrix} \quad (10)$$

$$\boldsymbol{I_{jk}} = \boldsymbol{m_i} \left(\boldsymbol{r_{jk}} \boldsymbol{L_i}\right)^2 \quad (11)$$

m_i and L_i (i = A, F, or H) denote the mass and length of segment i, respectively. The r_{jk} (j = X, Y, and Z, k = X, Y, and Z) coefficients are presented in Table 4.

Example based on specific segment lengths and body mass:

Table 5 Exemplary Mass, length, COM and inertia tensor for one subject

Male	Arm	Forearm	Hand
Mass (kg)	1.8	1.275	0.45
length(m)	0.33	0.26	0.20
COM x,y,z (m)	0.0056, -0.1482, -0.0085	0.0026, -0.1082, 0.0036	0.0026, -0.1082, 0.0036
Ixx (kg.m²)	0.0186	0.0067	0.0012
Iyy (kg.m²)	0.0038	0.0010	0.0005
Izz (kg.m²)	0.0198	0.0063	0.0010
Ixy (kg.m²)	0.0007	0.0001	0.0001
Ixz (kg.m²)	0.0005	0.0000	0.0001
Iyz (kg.m²)	0.0001	-0.0005	-0.0001
